# Glucocorticoid Signaling and the Aging Heart

**DOI:** 10.3389/fendo.2020.00347

**Published:** 2020-05-27

**Authors:** Diana Cruz-Topete, Robert H. Oakley, John A. Cidlowski

**Affiliations:** ^1^Department of Molecular and Cellular Physiology, Center for Cardiovascular Diseases and Sciences, LSU Health Sciences Center, Shreveport, LA, United States; ^2^Signal Transduction Laboratory, Department of Health and Human Services, National Institute of Environmental Health Sciences, National Institutes of Health, Durham, NC, United States

**Keywords:** glucocorticoids, aging, heart, cardiomyocytes, myocardium

## Abstract

A decline in normal physiological functions characterizes the aging process. While some of these changes are benign, the decrease in the function of the cardiovascular system that occurs during aging leads to the activation of pathological processes associated with an increased risk for heart disease and its complications. Imbalances in endocrine function are also common occurrences during the aging process. Glucocorticoids are primary stress hormones and are critical regulators of energy metabolism, inflammation, and cardiac function. Glucocorticoids exert their actions by binding the glucocorticoid receptor (GR) and, in some instances, to the mineralocorticoid receptor (MR). GR and MR are members of the nuclear receptor family of ligand-activated transcription factors. There is strong evidence that imbalances in GR and MR signaling in the heart have a causal role in cardiac disease. The extent to which glucocorticoids play a role in the aging heart, however, remains unclear. This review will summarize the positive and negative direct and indirect effects of glucocorticoids on the heart and the latest molecular and physiological evidence on how alterations in glucocorticoid signaling lead to changes in cardiac structure and function. We also briefly discuss the effects of other hormones systems such as estrogens and GH/IGF-1 on different cardiovascular cells during aging. We will also review the link between imbalances in glucocorticoid levels and the molecular processes responsible for promoting cardiomyocyte dysfunction in aging. Finally, we will discuss the potential for selectively manipulating glucocorticoid signaling in cardiomyocytes, which may represent an improved therapeutic approach for preventing and treating age-related heart disease.

## Introduction

Aging is characterized by a gradual decline in all physiological functions, a decrease in repair mechanisms, senescence, and eventually death. The endocrine system is a complex network system that regulates virtually all of an organism's biological processes, including development, growth, reproduction, metabolism, blood pressure, and responses to stressors (1, 2). Aging leads to significant alterations in the endocrine system, but imbalances in the endocrine system also affect the aging process (1–3). For example, the secretory patterns of hormones produced by the hypothalamic–pituitary axis are altered in the elderly population as well as the sensitivity to hormones by their target organs. Conversely, imbalances in the production of hormones or alterations in their negative feedback loops have been shown to accelerate the aging process by leading to disturbances in metabolism, cardiovascular function, and cognition (1, 2). Therefore, there is increasing interest in understanding the association between aging and endocrine function so that novel therapies can be developed to prevent/treat age-related endocrine disorders and the deleterious effects of endocrine imbalances during the aging process.

Aging is closely associated with alterations in cardiovascular function. Structural and functional changes in the heart and vasculature during aging are characterized by vascular stiffening, increased left ventricular wall thickness, hypertrophy, fibrosis, changes in maximal heart rate, and alterations in cardiac diastolic function (4–6). Age-related cardiac changes result from molecular alterations in the expression and function of proteins involved in maintaining cardiomyocyte structure, survival, calcium handling, redox balance, and metabolism (7). Additionally, changes in the expression profile of cardiac interstitial cells, including endothelial cells and fibroblasts, contribute to phenotypic changes in the aging heart (8). Although these alterations are not necessarily pathological in nature by themselves, they do increase the risk for cardiac damage and heart failure in the elderly population. There are several factors that further exacerbate the aging effect and make the “old heart” more likely to fail. For example, aging-associated alterations in inflammatory and fibrogenic pathways are exacerbated by metabolic imbalances, which are intertwined with a decline in endocrine function (9).

The relationship between the endocrine system, aging, and the cardiovascular system has been illustrated by the increased incidence of cardiovascular complications associated with the decline in gonadal hormone production. The risk of heart disease in women undergoing menopause is significantly higher than that in younger women (6, 10). Studies have suggested that the increased risk of heart disease in post-menopausal women is associated with a decline in ovarian hormone (estrogen and progesterone) production (11). Decreased ovarian function correlates with an elevation in the serum concentrations of atherogenic lipids (low-density lipoproteins and total cholesterol) and a decrease in the levels of cardioprotective lipids (high-density proteins) (12, 13). A decline in androgen production in males during aging has also been associated with higher cardiovascular risk due to increases in fat mass and the development of insulin resistance (12–14). The age-related decline in sex hormones has been considered one of the most clinically significant associations between the endocrine and cardiovascular systems. However, aging clearly influences other endocrine systems, and conversely, these endocrine alterations further influence the aging process. For example, with aging, glucose homeostasis is altered, which leads to an increased risk for metabolic complications such as type 2 diabetes that significantly elevate the risk for vascular and heart disease in the elderly population (2). Additionally, aging leads to changes in growth hormone (GH) and insulin growth factor 1 (IGF-1) levels. Alterations in GH and IGF-1 have profound effects on body composition and muscle strength, bone density, metabolism and the lipid profile, which in turn contribute to the deterioration of cardiac function (15–17). Therefore, a deeper understanding of the interactions between the endocrine system, aging, and the cardiovascular system is of great clinical interest, since it is estimated that more than 70% of individuals over 60 years of age suffer from cardiovascular diseases (3), and by 2050, ~17% of the population worldwide will be over 65 years of age. In the present review, we will discuss the effects of aging on the production and biological function of the primary stress hormones glucocorticoids and how alterations in glucocorticoid signaling affect the aging heart. We will also review the potential contribution of GR signaling to the vasculature morphological and functional changes that occur during the aging process. In addition, we will briefly review the cardiac effects of glucocorticoids signaling via MR in the heart. Finally, we will discuss whether the manipulation of glucocorticoid signaling in cardiomyocytes could prevent/revert aged-related heart disease.

## Mechanisms Regulating Glucocorticoid Secretion and Physiological Effects

### Regulation of Glucocorticoid Secretion

**Figure 1:**
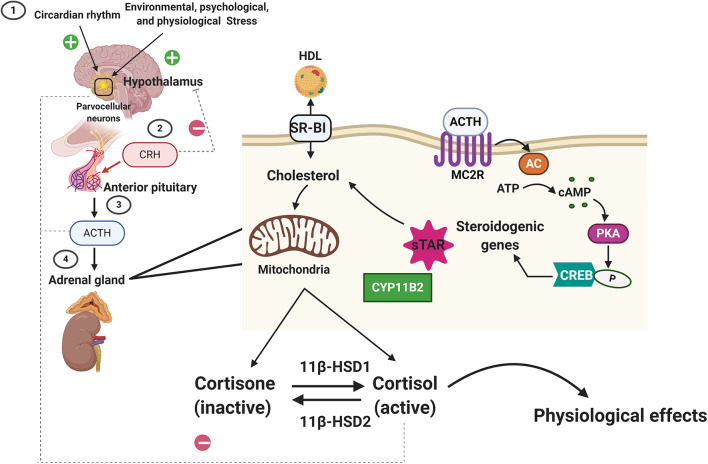
Regulation of glucocorticoid synthesis and secretion by the hypothalamic-pituitary-adrenal axis. Exposure to stressors and changes in our daily cycle (circadian rhythms) stimulate the parvocellular neurons within the paraventricular nucleus of the hypothalamus to release corticotropin releasing hormone (CRH). CRH then triggers the secretion of the adrenocorticotropic hormone (ACTH) from the anterior pituitary gland. ACTH then binds to its receptors (melanocortin receptor 2, MC2R) located on the cortex of the adrenal gland. ACTH binding to MC2R leads to the activation of adenylyl cyclase (AC) and the production of intracellular cyclic adenosine monophosphate (cAMP). cAMP then activates protein kinase A (PKA), which then phosphorylates cAMP response element-binding protein (CREB), which then promotes steroidogenic gene expression (Cytochrome P450 Family 11 Subfamily B Member, CYPB2, and the steroidogenic acute regulatory protein, StAR) that leads to the transport of cholesterol (imported from the blood into the cortical cells via the scavenger receptor type B class 1, SARB1) into the mitochondria, where glucocorticoids are synthesized (steroidogenesis) by the action of mitochondrial and smooth endoplasmic reticulum enzymes. The biologically active form of the glucocorticoid is the unbound cortisol that can be converted to the inactive form, cortisone by type 2 11β-hydroxysteroid dehydrogenase. Type 1 11β-hydroxysteroid dehydrogenase converts the cortisone to cortisol. Homeostasis in glucocorticoids synthesis and secretion is maintained by the negative feedback loop suppressing ACTH levels in the anterior pituitary and CRH levels in the hypothalamus.

Glucocorticoids (cortisol in humans; corticosterone in rodents) are steroid hormones produced by the zona fasciculata of the adrenal cortex in a circadian manner and in response to stress (Figure 1). Glucocorticoid secretion is regulated by the hypothalamic-pituitary axis (18, 19). Exposure to physical, psychological, and environmental stressors stimulates parvocellular neurons within the paraventricular nucleus of the hypothalamus to release corticotropin releasing hormone (CRH) at the median eminence into the capillary plexus of the hypothalamo-hypophyseal portal system (Figure 1). CRH is then carried to the anterior lobe of the pituitary gland, where it stimulates the production and secretion of adrenocorticotropic hormone (ACTH) by corticotroph cells (Figure 1). Once in circulation, ACTH binds G protein-coupled receptors located on the extracellular membranes of the zona fasciculata and zona reticularis of the adrenal cortex (20). ACTH binding to its receptors leads to the activation of adenylyl cyclase and the production of intracellular cyclic adenosine monophosphate (cAMP). Increased formation of cAMP triggers the activation of protein kinase A (PKA), which then phosphorylates and induces hormone-sensitive lipase to hydrolyze cholesteryl esters into cholesterol (21, 22). PKA also leads to the activation of the steroidogenic acute regulatory protein (StAR) (23), which then transports cholesterol into the mitochondria, where glucocorticoids are synthesized (steroidogenesis) by the action of mitochondrial and smooth endoplasmic reticulum enzymes (Figure 1).

Following synthesis, glucocorticoids are released from the adrenal glands and are bound to plasma proteins, in particular to corticosteroid-binding globulins (CBGs). Approximately 80% of circulating glucocorticoids are bound to CBG (24, 25). Glucocorticoids are released from their binding proteins by the action of neutrophil elastases at sites of inflammation (25, 26). Free glucocorticoids then diffuse through cell membranes. The cellular levels of glucocorticoids are controlled by 2 enzymes working in an opposing manner: 11β-hydroxysteroid dehydrogenase type 2 (11βHSD2) oxidizes cortisol into the inactive metabolite cortisone whereas 11β-hydroxysteroid dehydrogenase type 1 (11βHSD1) converts cortisone to cortisol (27). Once inside the cell, glucocorticoids bind their receptor, the glucocorticoid receptor (GR, NR3C1). GR is a member of the nuclear receptor family of ligand-activated transcription factors and is ubiquitously expressed in all nucleated cells throughout the body. Glucocorticoids can also bind the closely related mineralocorticoid receptor (MR, NR3C2) which has a more restricted tissue distribution than GR (28). In most cell types, MR appears to be principally bound by aldosterone due to the action of 11βHSD2. However, glucocorticoid occupancy of MR can occur in certain tissues, such as the heart, that are deficient in this enzyme. In the face of an acute stressor, the increase in glucocorticoid levels and their signaling via GR is beneficial and aids the body in restoring homeostasis by modulating the immune response, metabolism, and cardiovascular function (Figure 1). However, exposure to chronic stress or imbalances in glucocorticoid synthesis and secretion (e.g., Cushing's disease or Addison's disease) leads to an array of pathologies, ranging from immune disorders to metabolic and cardiovascular complications (29). Therefore, in normal physiology, glucocorticoid levels are tightly regulated by a negative feedback loop at the level of the hypothalamus and pituitary gland, the availability of CBG in circulation and at target tissues by the action of 11βHSD1 and 11βHSD2 (26).

As elegantly discussed by McEwen BS (30), depending on the context, glucocorticoids can exert both protective and deleterious effects on the body. An acute increase in glucocorticoids in response to stress is critical to maintaining homeostasis and allostasis (survival) (30). However, imbalances in glucocorticoid secretion patterns and levels due to exogenous administration (increased levels) or pathological states (overproduction or deficiency) can lead to or accelerate disease processes, including metabolic and cardiovascular complications (30), which are commonly found in the elderly. During the aging process, glucocorticoid secretion patterns undergo several modifications characterized by impairments in their circadian profile. While cortisol increases early during the day in the young, a flattening in glucocorticoid rhythm is seen in a subpopulation of the elderly in particular on those suffering from chronic disease, including cognitive impairments such Alzheimer's disease (2, 31, 32). This dysregulation in natural glucocorticoid rhythm seems attributable to a reduction in sensitivity to hypothalamic-pituitary-adrenal (HPA) axis negative feedback control due to an increased glucocorticoid level (32). However, there is no cause and effect relationship established between HPA axis dysregulation and cognitive impairments, dementia, depression, anxiety, as well as an increased risk of Alzheimer's disease, diabetes, and hypertension in the elderly (33–36). Studies are needed to fully establish if there is a physiological effect between the HPA axis dysregulation and increased glucocorticoid levels and the risk for cognitive impairments, anxiety, depression, and chronic inflammatory disorders in the elderly.

### Glucocorticoid Receptor Signaling and Physiology

The GR is encoded by the NR3C1 gene located on chromosome 5 (5q31) in humans and chromosome 18 in mice. The GR gene is composed of 9 exons, and three domains comprise the GR protein: (1) an amino-terminal transactivation domain (NTD), encoded by exon 2, which mediates some interactions with co-regulators and the transcriptional machinery; (2) a DNA binding domain (DBD), encoded by exons 3 and 4, which contains two zinc-finger motifs involved in genomic interactions; and (3) a ligand-binding domain (LBD), encoded by exons 5–9, that contains a hydrophobic pocket for glucocorticoid binding and an activation function (AF2) that interacts with transcriptional coregulators. The DBD and LBD are separated by a region known as the hinge region that is involved in receptor dimerization. Additionally, there are two nuclear localization signals, NL1 and NL2, located in the DBD/hinge region junction and within the LBD, respectively. Although only one gene encodes GR, alternative splicing in exon 9 results in two receptor isoforms, GRα and GRβ. GRα is thought to mediate the majority of physiological actions of glucocorticoids; however, recent studies suggest that GRβ can also regulate the expression of numerous genes involved in inflammation (37) at the level of transcription. In addition to these most abundant isoforms, the GR gene can also give rise to three additional splice isoforms known as GRγ, GR-A, and GR-P. Furthermore, GRα can also undergo alternative translation in exon 2, leading to the formation of eight additional GR isoforms, GRα: GRα-A, GRα-B, GRα-C1, GRα-C2, GRα-C3, GRα-D1, GRα-D2, and GRα-D3. These isoforms exhibit unique expression patterns and gene regulatory profiles and are thought to play an important role in the tissue-specific actions of glucocorticoids (38).

An inactive GR is located in the cytoplasm in complex with chaperone proteins (hsp90, hsp70, and p23) and immunophilins of the FK506 family (FKBP51 and FKBP52) (38–40). Upon hormone binding, GR undergoes a conformational rearrangement that results in the dissociation of the associated proteins and the exposure of two nuclear localization signals that trigger GR rapid translocation into the nucleus (38). Once in the nucleus, GR exerts effects on the expression of target genes by directly binding to DNA or by interacting with other transcription factors (genomic mechanisms) (29, 38, 41, 42). Additionally, GR can act via protein-protein interactions in the cytoplasm (non-genomic mechanisms) that lead to rapid cellular responses that trigger the activation of various kinases, including PI3K, AKT, and MAPKs (43–45).

In addition to the different GR isoforms and genomic and non-genomic mechanisms of action, post-translational modifications (PTMs) affect the transcriptional and physiological activity of GR. The effects of phosphorylation have been extensively characterized and have been found to be critical for GR genomic and non-genomic interactions, including cellular localization, half-life, and interaction with DNA and coregulators. Additional PTMs, including ubiquitination, acetylation, and sumoylation, have been shown to influence GR activity. The GR gene and protein structure, isoforms, PTMs, and mechanisms of signaling are described in detail in recent reviews by Oakley et al. (38) and Scheschowitsch et al. (46).

**Figure 2:**
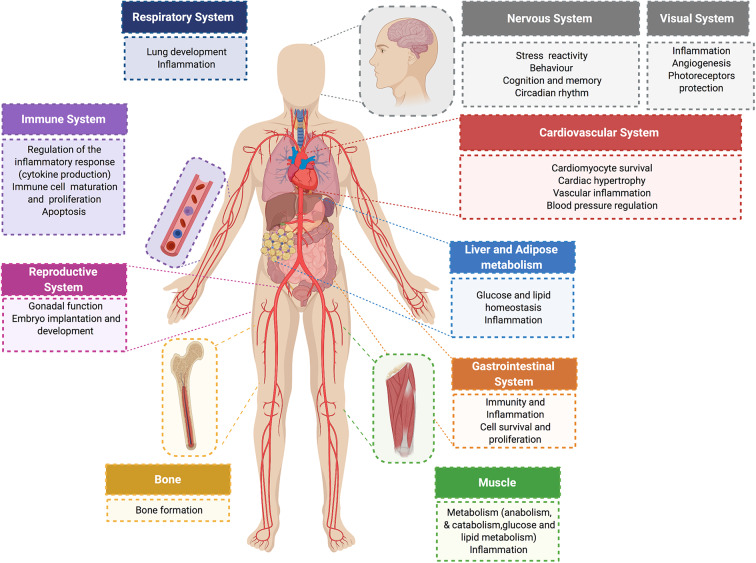
Physiological glucocorticoid effects. Glucocorticoids exert direct actions in major organ systems of the human body. Their effects range from modulation of the inflammatory and immune response to the regulation of glucose and lipid metabolism in different tissues. Glucocorticoid signaling plays an essential role on the cardiovascular system. In normal physiology, glucocorticoids exert cardioprotective and anti-inflammatory effects, and have an essential role in controlling blood pressure and cardiac function.

Further complexity and diversity in the physiological responses of glucocorticoids are achieved by these hormones acting in a sex- and tissue-specific manner (47–50), and by glucocorticoid activation of MR in cells deficient in 11βHSD2 Like GR, MR is comprised of an NTD that regulates transcriptional activity, a DBD for interacting with specific genomic sequences, and a LBD that binds glucocorticoids (or aldosterone) (51). The DBD of MR is 94% identical to the DBD of GR. Therefore, in response to glucocorticoids, GR and MR can bind the same DNA response element but these receptors elicit distinct transcriptional effects on target genes (52, 53). Figure 2 summarizes some of the effects of glucocorticoids and GR in different organ systems.

How aging alters the molecular mechanisms of glucocorticoid signaling remains largely unknown. A study by Murphy et al. (54) found that intracellular GR trafficking is impaired in the aging hippocampus due to a deficit in chaperone proteins, which diminish GR signaling within this area of the brain. A later study showed that GR mRNA levels in the cortex of the brain rise between infancy and adolescence and decline between adulthood and advanced age (55). The same study found that GR mRNA levels remain stable across the life span in the hippocampus (55). However, the mechanism that causes this aged-related change in GR expression in the cortex is unknown.

Little is also known about the effects of aging on GR signaling in the cardiovascular system. For example, no studies have been performed to test whether the expression of GR isoforms changes with age or whether there are alterations in GR phosphorylation or in other PTMs that affect GR cellular signaling. Additionally, no data exist on how activation of GR signaling by exposure to acute or chronic stressors alters the gene expression profile of the aging heart. In the next section, we will discuss the latest research on glucocorticoids and GR signaling in the heart and the potential interplay between age and stress in cardiac health.

## Glucocorticoid Signaling and the Aging Cardiovascular System

### Glucocorticoids and the Vasculature in Aging

Several mechanisms have been shown to contribute to vascular pathology in aging. However, oxidative stress [increased reactive oxygen species (ROS) production] is a significant contributor to coronary artery disease, myocardial ischemia, and stroke in the elderly (56). There is strong evidence that endothelial dysfunction caused by ROS leads to both impaired dilation of coronary arteries and a pro-atherogenic vascular phenotype in aging (57–61) by altering endothelium nitric oxide (NO) production (62), which is a major endogenous vasodilator, anti-inflammatory, and anti-thrombotic molecule (59–61). The decreased in NO synthesis has been proposed to be responsible for many of the age-associated vasoconstriction and reduced tissue perfusion (63–66). NO bioavailability during aging is also affected by alterations in endothelial nitric oxide synthase (eNOS) activation status, concentrations of L-arginine (NO substrate) and decreasing expression of guanosine triphosphate cyclohydrolase 1 (GTPCH1) mRNA, which is the rate-limiting enzyme in the production of the cofactor tetrahydrobiopterin (BH4) (59–61, 67). Glucocorticoids exert effects on the vasculature by GR regulation of a vast array of signaling pathways that are involved in development, angiogenesis, oxidative stress, and inflammation in vascular smooth and endothelial cells (59, 68). Among the most well-characterized effects of glucocorticoids on the vasculature are those mediated by GR modulation of NO biosynthesis (68, 69). NO production is elevated in the early response to glucocorticoids, but its levels are significantly repressed at later phases of the stress response, or when exposed to sustained increases in systemic glucocorticoids (70). Studies using mouse models have shown that systemic glucocorticoid administration leads to hypertension by a mechanism involving inhibition of NO metabolites, NO2–, and NO3– (indicators of total NO levels), and by downregulating the gene expression of NO synthase III in endothelial cells (71). More recent studies on transgenic mouse models showed that these effects are mediated directly by vascular smooth muscle and endothelial cells GR expression (72, 73). Although mice lacking GR in vascular smooth muscle cells displayed a normal phenotype under basal physiology, when glucocorticoids were acutely administered, the smooth cell-specific knockout mice were protected from glucocorticoid-induced hypertension as compared to their littermate controls (72). These results suggest a role for smooth cells-GR in regulating the hypertensive response *in vivo* (72). An intact GR signaling seems to play a more critical role in vascular endothelial cells. Mice lacking GR in endothelial cells were relatively resistant to dexamethasone-induced hypertension (74) and displayed increased expression of eNOS and inducible nitric oxide synthase (iNOS), which are critical enzymes in NO synthesis (73, 74). Endothelial cell GR knockout mice also have an exacerbated hypotensive response after lipopolysaccharide (LPS) administration due to increase NO production by endothelial cells (74).

In addition to the effects of glucocorticoids on NO biosynthesis, glucocorticoids can increase the expression of angiotensin II type I receptors in smooth muscle cells affecting blood pressure (75). Moreover, glucocorticoids can influence the influx of Na+ and Ca2+ into vascular smooth muscle affecting contractility and therefore leading to alterations in blood pressure. However, this effect could be mediated by the closely related mineralocorticoid receptor (MR). In addition, glucocorticoids exert effects on the vascular tone regulation by inhibiting angiogenesis, cell proliferation, viability, and cell migration via GR regulation of the anti-angiogenic thrombospondin-1 (74, 76). Moreover, glucocorticoids have indirect effects on the vasculature throughout their actions on inflammatory cells within the vasculature that contribute to endothelial cell responses and function. In the context of aging, the role of GR signaling in the vasculature has not been studied or characterized. However, based on the data discussed above, glucocorticoid signaling is most likely influencing the aged vasculature, and depending on the context, the effects may be beneficial or detrimental. Future studies are needed to fully elucidate the role of GR signaling in the vasculature in aging, and whether pharmacological regulation of this signaling pathway can be potentially used to control hypertension in the elderly.

### Glucocorticoids and the Heart

In the last decade, studies have shown that depending on the physiological context (e.g., sex, disease state, etc.), type and duration of the stress (e.g., environmental, psychological, acute or chronic), and mechanisms of signaling (via GR or the closely related MR) glucocorticoids have many effects on the heart. Some of these effects are positive and essential for life, whereas other effects can be detrimental for cardiac health. Clinical data suggest that decreased systemic GR signaling is associated with a reduction in cardiac contractile force, systolic dysfunction, coronary artery disease, dilated cardiomyopathy, and progression to heart failure (77–81). Similarly, overactivated glucocorticoid signaling has been shown to lead to negative cardiac outcomes. For example, prenatal exposure to glucocorticoids due to increased stress levels during pregnancy increases the risk for developing cardiovascular disease in adulthood (82). Additionally, excessive glucocorticoid levels due to endocrine disorders or pharmacological treatment are linked to major risk factors for cardiovascular disease, including metabolic syndrome and hypertension, and to pathological cardiac hypertrophy and failure (83–86). A limitation of many these studies is that they do not distinguish between systemic actions of glucocorticoids and direct local actions of glucocorticoids on the heart and the vasculature.

Both *in vitro* and *in vivo* models have provided significant insights into the direct role played by cardiomyocyte GR. Since global deletion of GR leads to death soon after birth (87), Rog-Zielinska et al. performed prenatal studies on mice with global knockout of GR and on mice with conditional knockout of GR in cardiomyocytes and vascular smooth muscle cells to investigate the role of glucocorticoid signaling in fetal heart maturation (88). Their studies showed that prenatal inactivation of global GR decreases the size of the heart, impairs diastolic function, and alters cardiomyocyte structure and fibril organization. Hearts from these mice also presented with defects in the levels of alpha myosin heavy chain (Myh6), the major contractile protein in adult hearts, and in calcium handling proteins, including the cardiac ryanodine receptor (Ryr2), sodium calcium exchanger (NCX1), and the sarcoplasmic/endoplasmic reticulum calcium ATPase (SERCA2a) (88). These findings highlight the role of glucocorticoid signaling in the development of the heart, particularly in regulating the maturation and expression of genes critical for cardiomyocyte architecture and function (88).

The role of GR signaling in the postnatal heart has also been demonstrated with transgenic mouse models. An elegant study by Sainte-Marie et al. (89) demonstrated that cardiomyocyte overexpression of human GR in the mouse heart leads to an abnormal heart rhythm (bradycardia) and electrical deficits in the heart, including an extended PQ interval, long QRS duration, and increased QTc dispersion (89). *In vitro* studies using cardiomyocytes isolated from the heart of these transgenic mice showed that the observed phenotype resulted from defects in sodium and potassium currents and increases in L-type calcium currents, calcium transient amplitudes, and the sarcoplasmic reticulum (SR) calcium content (89). These results suggested that overactivation of GR signaling in cardiomyocytes leads to abnormalities in the cardiac conduction system, but no structural abnormalities were found in this mouse model.

Studies by Oakley et al. (90) on mice lacking GR only in cardiomyocytes showed that inactivation of cardiomyocyte GR leads to premature death (median survival age is ~7 months) due to the development of systolic dysfunction and dilated cardiomyopathy. In agreement with the prenatal and overexpression findings, this study also found that cardiomyocyte GR plays a role in the regulation of genes involved in cardiac structure and calcium handling (90). Interesting the deleterious effects of GR inactivation occur earlier and exacerbated in males compared to females (91).

**Figure 3:**
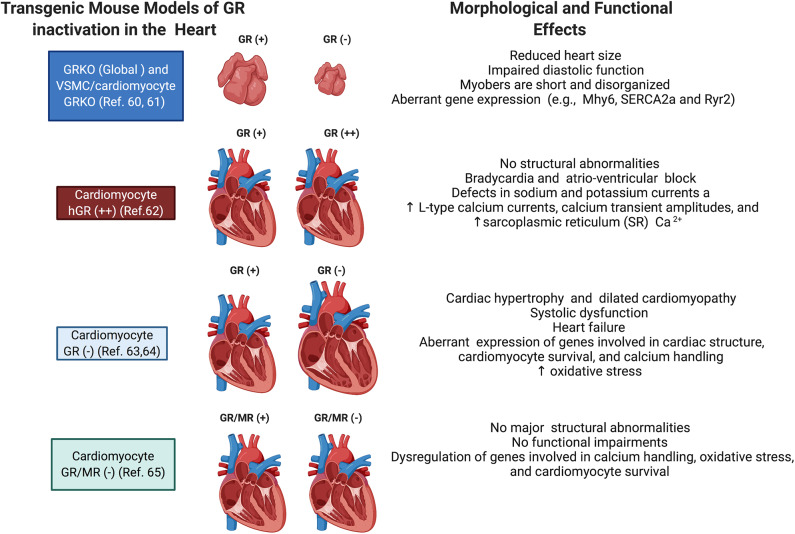
Schematic representation of the phenotypes of transgenic mice targeting GR in cardiomyocytes. Shown are the major morphological and functional phenotypes associated with GR overexpression (GR+), GR inactivation (GR–), and GR and MR inactivation (GR/MR–) in the whole heart, vascular smooth muscle cells (VSMC), and cardiomyocytes.

Recent studies by the same group using a transgenic mouse model lacking both GR and MR in cardiomyocytes showed that inactivation of MR rescued the left ventricular dysfunction and premature mortality observed in the absence of GR (92). Despite exhibiting a reduction in several Ca2+ handling genes as well as increased expression of pathological cardiac hypertrophy markers, the double knockout mice have normal heart morphology and function compared to the single GR knockout mouse model (92). These data suggest that under conditions of myocardial stress, such as that triggered by inactivation of cardiomyocyte GR, MR signaling becomes deleterious and promotes cardiac disease (93–96). Cardiac MR signaling has also been shown to be harmful in other models of heart dysfunction. Evidence suggests that pathological conditions resulting in the accumulation of reactive oxygen species may be responsible for the inappropriate gene regulatory activity of glucocorticoid occupied MR in the heart (97–99). Currently, there is much interest in cardiac MR signaling since treatment with the MR antagonists eplerenone or spironolactone leads to a reduction in morbidity and mortality in heart failure patients (100). The role of MR signaling in the heart is described in detail in recent reviews by Oakley et al. (78) and Young et al. (101). Figure 3 summarizes some of the effects of glucocorticoids on the heart.

Regarding the role of glucocorticoids in the aging process, some studies show that an increase in glucocorticoid levels accelerates the aging process and increases the risk of premature mortality due to negative effects on vasculature, adipose tissue, and lipid and carbohydrate metabolism (102–104). There are no studies, however, on the direct effects of glucocorticoid signaling on aging cardiomyocytes and the heart. Therefore, it is still unclear whether increased glucocorticoid levels directly contribute to cardiovascular complications during aging or whether the interplay of glucocorticoid signaling with additional risk factors, such as obesity, hypertension, and diabetes, drives the negative systemic actions of these hormones on the heart. In the next section, we will discuss the potential role of glucocorticoids and GR signaling in cardiomyocyte dysfunction in aging and whether manipulation of this system could be potentially used to maintain a healthy heart.

### Glucocorticoids and Cardiomyocyte Dysfunction in Aging

Cardiac hypertrophy is a hallmark of aging and a major cause of heart failure (3, 4, 105, 106). Increased glucocorticoid levels have been associated with pathological cardiac hypertrophy (107). *In vitro* and *in vivo* studies show that glucocorticoid administration under non-stress conditions leads to cardiomyocyte hypertrophy characterized by cellular structural and morphological changes and an increase in the levels of the cardiac hypertrophy markers atrial natriuretic factor (ANF), β-myosin heavy chain (MHC), and skeletal actin (SKA). Additionally, changes in the expression of the pro-hypertrophic genes α1b-adrenergic receptor (ADRα1b), insulin growth factor (IGF)-IR), and interleukin (IL)-6R were found in response to glucocorticoid treatment (108–110). IGF-I signaling is one of the best characterized pathways that is known to be involved in regulating lifespan in animal models (111). Decreased activation of this signaling has been associated with improved cardiomyocyte performance and an attenuation of age-related structural changes in cardiomyocytes (112, 113); however, studies in humans have shown a correlation between low IGF-I levels and an increased risk for heart failure in elderly patients (114). Additionally, an increase in circulating levels of IGF-I resulting from growth hormone (GH) therapy has been shown to be beneficial for the treatment of heart failure patients (115–117). IGF-I signaling has been proposed to exert this cardioprotective effect on aging by decreasing reactive oxygen species production by the mitochondria and thereby reducing cellular oxidative stress (118, 119). Therefore, some of the effects of glucocorticoids on the aging heart may be mediated by GR-dependent regulation of this pathway. Future studies are needed to further elucidate the relevance of the crosstalk between GR and IGF-I signaling in the context of heart aging and heart failure.

Under stressful conditions (e.g., starvation), studies show that glucocorticoids protect cardiomyocytes from cell death through GR-dependent upregulation of anti-apoptotic proteins such as Bcl-xL and repression of pro-apoptotic proteins, such as Gas2 (108). These results suggest that the elevation in glucocorticoid levels during aging may have a direct protective role in the heart by preventing cardiomyocyte death triggered by age-related changes in the levels of proteins associated with cardiomyocyte architecture and mechanical properties (e.g., myosin heavy chain and sarcomeric actin isoforms), calcium handling [e.g., SERCA2 [SR (sarcoplasmic reticulum)/ER (endoplasmic reticulum) Ca2+-ATPase 2]], and DNA repair mechanisms (120).

Collectively, these findings suggest that glucocorticoids can both positively and negatively influence the function of the heart through direct effects on cardiomyocytes. Future research needs to be focused on elucidating the mechanisms by which glucocorticoids exert these positive/negative actions on cardiomyocytes, in which context glucocorticoids signal through GR and/or MR, and whether the majority of their beneficial effects on the heart are mediated by cardiomyocyte GR signaling or MR signaling.

## Conclusions and Future Perspectives

Aging has a significant impact on the endocrine system, affecting hormonal secretion patterns and hormone sensitivity, but the endocrine system also modulates aging by triggering changes in gene expression and the cellular structure of different organ systems. There is a clear connection between the endocrine system and cardiac health in aging. Whether this connection leads to positive or negative effects depends on the influence of intercurrent chronic diseases, nutritional status, and other age-related changes. In the context of the heart, the aging process is associated with significant morphological and functional alterations, including cardiac hypertrophy and fibrosis, and changes in end-systolic and diastolic volume, and cardiac filling pressure and diastolic relaxation, among other parameters. The aging heart also displayed marked abnormalities in the cardiac conduction system that impacts the heart electrical properties (6, 121). Given the role of GR signaling in regulating the expression of key genes involved in Ca^2+^ handling, cell death pathways, and cardiac hypertrophy and fibrosis, future studies are needed to test if targeting cardiomyocyte activation of GR with high affinity agonists holds promise for the development of new therapies for combatting cardiac disease in the elderly.

## Author Contributions

All authors listed have made a substantial, direct and intellectual contribution to the work, and approved it for publication.

## Conflict of Interest

The authors declare that the research was conducted in the absence of any commercial or financial relationships that could be construed as a potential conflict of interest.
